# Metal-Assisted Chemical Etching and Electroless Deposition for Fabrication of Hard X-ray Pd/Si Zone Plates

**DOI:** 10.3390/mi11030301

**Published:** 2020-03-13

**Authors:** Rabia Akan, Thomas Frisk, Fabian Lundberg, Hanna Ohlin, Ulf Johansson, Kenan Li, Anne Sakdinawat, Ulrich Vogt

**Affiliations:** 1 KTH Royal Institute of Technology, Department of Applied Physics, Biomedical and X-ray Physics, Albanova University Center, 106 91 Stockholm, Sweden; 2 MAX IV Laboratory, Lund University, 22 100 Lund, Sweden; 3 SLAC National Accelerator Laboratory, 2575 Sand Hill Road, Menlo Park, CA 94025, USA

**Keywords:** X-ray diffractive optics, zone plate, high aspect ratio nanostructures, metal-assisted chemical etching, electroless deposition

## Abstract

Zone plates are diffractive optics commonly used in X-ray microscopes. Here, we present a wet-chemical approach for fabricating high aspect ratio Pd/Si zone plate optics aimed at the hard X-ray regime. A Si zone plate mold is fabricated via metal-assisted chemical etching (MACE) and further metalized with Pd via electroless deposition (ELD). MACE results in vertical Si zones with high aspect ratios. The observed MACE rate with our zone plate design is 700 nm/min. The ELD metallization yields a Pd density of 10.7 g/cm${}^{3}$, a value slightly lower than the theoretical density of 12 g/cm${}^{3}$. Fabricated zone plates have a grid design, 1:1 line-to-space-ratio, 30 nm outermost zone width, and an aspect ratio of 30:1. At 9 keV X-ray energy, the zone plate device shows a first order diffraction efficiency of 1.9%, measured at the MAX IV NanoMAX beamline. With this work, the possibility is opened to fabricate X-ray zone plates with low-cost etching and metallization methods.

## 1. Introduction

X-ray microscopy is a powerful scientific tool used for the study of different samples in a variety of disciplines [1,2]. Especially in the hard X-ray regime (multi-keV photon energies), X-ray microscopy offers the possibility to study thick samples with nanoscale resolution. Diffractive zone plate optics are commonly used as focusing and imaging optics in these X-ray microscopes. Zone plates are circular, dense grating structures with radially decreasing zone widths [3,4]. The outermost zone width defines the zone plate resolution, whereas the zone thickness defines the zone plate diffraction efficiency. As an example, to focus X-rays with 9 keV photon energy with maximum efficiency performance, a zone plate made from Pd would require a thickness of 2.5 μm. The required high aspect ratios at very small zone widths make hard X-ray zone plates challenging to fabricate [5,6]. The performance of hard X-ray microscopes is therefore often limited by the fabrication quality of the focusing optics.

Two main high aspect ratio zone plate fabrication routes have been presented in the literature. The first is direct electron-beam patterning either of an organic or inorganic resist mold. The mold is then filled with a metal by electrodeposition [7,8] or covered with a metal layer by atomic layer deposition [9,10]. The second method transfers the zone plate pattern into a metal layer by deep reactive ion etching [11,12]. However, in both cases, the achievable aspect ratios are limited to about 20:1. Attempts have been made to overcome this limit by stacking of multiple zone plates, either mechanical [13,14] or via multi-level e-beam exposures [15,16], which is very challenging.

Metal-assisted chemical etching (MACE) has recently been used as an alternative fabrication method to transfer a zone plate pattern into a Si substrate with the advantage of vertical etching and the possibility to reach high aspect ratios [17,18,19,20]. MACE is a wet-chemical process where a noble metal layer, such as a Au zone plate pattern, is transferred into a Si substrate by an etching solution consisting of a strong oxidizing agent and HF [21,22,23]. The Si is predominantly oxidized where it is in contact with the noble metal layer, which catalyzes the oxidation process. The HF subsequently dissolves the formed SiO${}_{2}$ [24,25].

Due to its low X-ray diffraction efficiency in the hard X-ray regime, the MACE processed Si zone plates are not suitable to be used directly as optical devices. Instead, they can be used as molds for high-Z materials, such as Pt, deposited with atomic layer deposition (ALD) [17,18]. Unfortunately, ALD is a rather slow and expensive process, and the homogeneous filling of deep and small trenches is challenging. Electrodeposition (ED) is an alternative wet-metallization process that is often used with polymer resist-based zone plate molds on conductive substrates [26]. ED is, however, challenging for metallizing MACE Si molds due to contacting difficulties to the Au layer at the bottom of the zone plate pattern. To overcome the challenge of contacting, the autocatalytic process of electroless deposition (ELD) is an alternative method to metalize the Si-based zone plates. In ELD, a metal complex is selectively reduced at a conductive surface [27,28]. The deposited metal now serves as a catalyst in the deposition reaction, ensuring continuous buildup of the metal.

In this work, we describe the fabrication process of hard X-ray Pd/Si zone plates using a simple and low-cost wet-chemical approach. MACE is used to transfer a zone plate pattern into a Si substrate, and ELD is used to metalize the Si zone plate mold with Pd. The Au zone plate pattern serves as a catalyst in both processes, under which the Si is selectively etched and on which the Pd is selectively deposited. Here, Pd is chosen as a high-Z material owing to an acceptable diffraction efficiency in the hard X-ray regime and the commercial availability of a well-formulated, stable ELD bath.

## 2. Materials and Methods

### 2.1. Materials

*p*-type, boron-doped Si (100) wafers with 1–5 Ωcm resistivity were purchased from Si-Mat. CSAR 62, amyl acetate, and dimethyl succinate were purchased from Allresist. Hydrofluoric acid (HF, 40%) and acetone were procured from Merck. Hydrogen peroxide (H${}_{2}$O${}_{2}$, 31%) and isopropanol were from D-BASF. The PD-Tech PC electroless Pd system was purchased from Atotech. Ethanol and *n*-pentane were from VWR.

### 2.2. Zone Plate Patterning Using Electron Beam Lithography (EBL)

**Figure 1:**
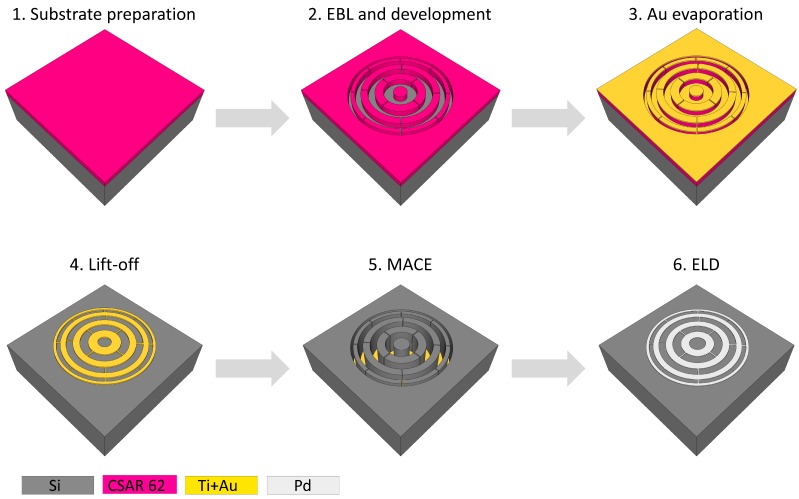
Schematic of the zone plate fabrication process.

The overall zone plate fabrication schematic is presented in Figure 1. The Si-wafers were cut into 1.5 × 1.5 cm${}^{2}$ substrates and pre-cleaned under sonication in acetone followed by isopropanol for 5 min each. Thereafter, the substrates were cleaned in oxygen plasma for 5 min in an Oxford Instruments PlasmaLab 80 Plus RIE/ICP system. Seventy nanometer CSAR 62 EBL resist was spin-coated on the substrates and baked on a 180 °C hot plate for 60 s. The zone plate patterning was performed using a 50 kV Raith Voyager EBL system. The written zone plate pattern had a diameter of 150 μm, an outermost zone width of 30 nm, and a line-to-space ratio of 1:1. The exposed CSAR 62 was developed in amyl acetate for 60 s at room temperature. Further, the substrates were rinsed in isopropanol and n-pentane for 10 s and 15 s, respectively, and descummed in an oxygen plasma for 13 s for removal of exposed resist residues. Using an in-house Eurovac/Thermionics electron beam evaporation deposition system, a 2 nm adhesive Ti layer followed by a 10 nm Au layer were evaporated at a rate of 1 Å/s. The resist lift-off was performed in dimethyl succinate under sonication for 10 min, and the substrates were thereafter rinsed in isopropanol and deionized (DI) H${}_{2}$O. The resulting substrates with Au zone plate patterns were dried under nitrogen gas.

### 2.3. MACE Processing of Si Zone Plate Molds

The Au patterned substrates were cleaned in oxygen plasma for 3 min right before MACE. The MACE zone plate processing was performed as reported previously [20]. Briefly, a 15 mL etching solution consisting of 0.68 M H${}_{2}$O${}_{2}$, 4.7 M HF, and 51 M DI H${}_{2}$O was prepared in a polytetrafluoroethylene bath. The clean substrates were immersed in the etching solution, and the MACE process was performed at room temperature for 75 s under light protection. The substrates were rinsed in copious amounts of DI H${}_{2}$O, transferred to ethanol, and dried in a Leica EM CPD300 critical point dryer.

### 2.4. Pd ELD Metallization of Zone Plates

The substrates were cleaned prior to ELD metallization in oxygen plasma for 3 min. This plasma oxidation step was found necessary for preventing Pd deposition on other sites than the catalytic Au layer at the bottom of the zone plate structures. A 100 mL plating solution consisting of 75 mL DI H${}_{2}$O, 15 mL PD-Tech PC Reduction Solution, and 10 mL PD-Tech PC Plus Make-Up Solution was prepared as specified by Atotech and heated to 40 °C. After 30 min of temperature stabilization, the Si substrates were vertically immersed in the plating solution, and the ELD proceeded for 30 min under stirring. After completion of the ELD, the substrates were rinsed in DI H${}_{2}$O and dried under nitrogen gas.

### 2.5. Characterization

Micrographs were obtained and cross-sections prepared with an FEI NOVA 200 dual-beam scanning electron microscopy (SEM) and focused ion beam (FIB) system.

The static contact angle measurements were performed using a Biolin Scientific Theta Lite Optical Tensiometer on 10 nm Au-coated Si substrates before and after a 3 min oxygen plasma treatment. At room temperature, a 5 μL DI H${}_{2}$O droplet was released on the substrate, and the contact angles on both sides of the droplet were continuously recorded. The presented data were the average value of the left and right contact angles over a 4 s contact time.

The Pd density was determined gravimetrically using a Sartorius analytical scale and a KLA Tencor P-15 surface profiler by weighing and measuring the thicknesses of several Pd films plated on 10 nm Au-coated Si plating bases.

The zone plate diffraction efficiency was quantified at the NanoMAX beamline at the MAX IV synchrotron radiation facility [29]. Several zone plates were illuminated by a coherent beam with a photon energy of 9 keV. A 500 μm aperture with a 25 μm-wide and 25 μm-thick tungsten central stop was placed before a zone plate, and a 10 μm order sorting aperture (OSA) was placed slightly upstream of the zone plate focal plane. The first order diffraction was recorded by a Crycam X-ray camera from Crytur. For the calculation of the first order diffraction efficiency, several images with the first order zone plate cone and the empty beam without the zone plate and OSA (but transmitted through the Si substrate) were recorded.

## 3. Results and Discussion

### 3.1. Zone Plate Fabrication

**Figure 2:**
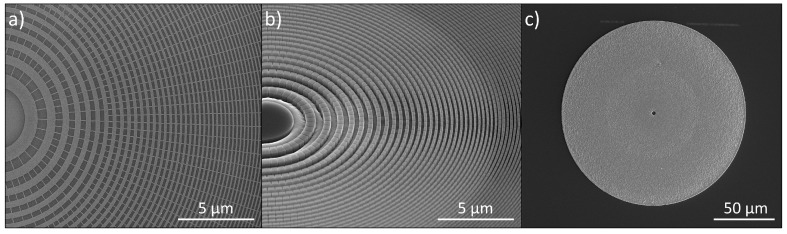
SEM micrographs of (**a**) Au patterned Si substrate (top-view), (**b**) MACE processed Si zone plate mold (52°-tilt view), and (**c**) ELD metalized Pd/Si zone plate showing selective plating in the zone plate area only (top-view).

As reported previously, a Au zone plate catalyst design with interconnects between the zones plate rings is essential to get a controlled, vertical etching during MACE at ambient processing conditions (Figure 2a) [20]. While zone verticality is ensured with this design, too thick zones will tilt due to mechanical instabilities. This is especially visible at the outermost parts of the zone plate where the zone widths are the smallest. Therefore, the deepest zone thickness was chosen as 900 nm in this study, giving an aspect ratio of 30:1 (Figure 2b). For 150 μm zone plates with a 10 nm Au catalyst layer, the observed MACE rate was 700 nm/min.

Measurements revealed a decrease of the contact angle for the oxygen plasma-treated Au films, indicating a more hydrophilic surface character and easier wetting. The measured contact angles before and after the oxygen plasma treatment were 89° and 29°, respectively. In addition, no plating was observed on sites other than on the Au at the bottom of the Si zone plate mold (Figure 2c). This suggested that the short plasma treatment was enough to grow a passivating oxide layer on the Si substrate. Thirty nanometer wide zones with thicknesses of 3 μm were evenly plated, and no limitations were observed with the Pd ELD process. These results are however not shown here due the stability issues of the Si nanostructures, resulting in tilted zones.

**Figure 3:**
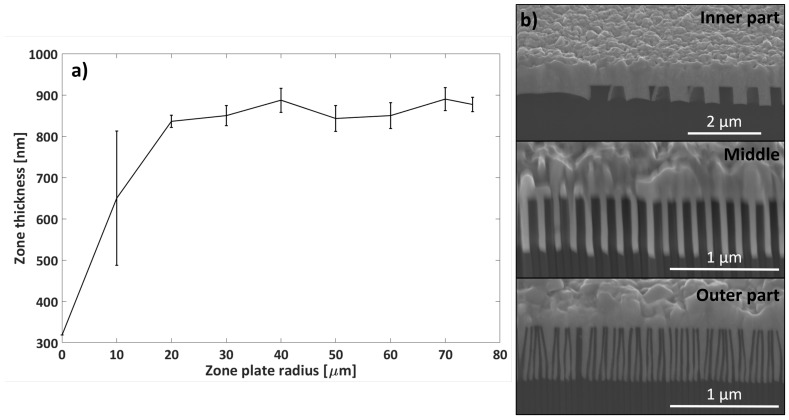
(**a**) Zone thickness profile over the zone plate radius based on image analysis of SEM cross-section micrographs. The error bars represent the zone thickness variation of the surrounding zones at each point. (**b**) SEM micrographs of the cross-sections of the inner, middle, and outer parts of a typical Pd/Si zone plate (52°-tilt view).

The decreasing zone widths resulted in a variation of zone thickness over the zone radius. The inner zones were wider than the outer zones and consequently relatively thin compared to the outer zones. In order to fill the zones fully with Pd via ELD, over-plating was unavoidable. The variation of zone thickness over the zone plate radius is presented in Figure 3a. The zone thickness variation was largest from the zone plate center to 20 μm outwards. However, this variation was not a big concern for the final zone plate performance since, normally, a central stop is used. The zone thickness was uniform from 20 μm to the outermost part of the zone plate, with a standard deviation of about ±30 nm. Cross-section SEM micrographs are presented in Figure 3b for the inner, middle, and outer parts of a typical Pd/Si zone plate. The tendency of non-vertical zones is visible for the 30 nm wide zones in the outer part, where the Si structures are slightly slanted.

### 3.2. Pd Density

**Figure 4:**
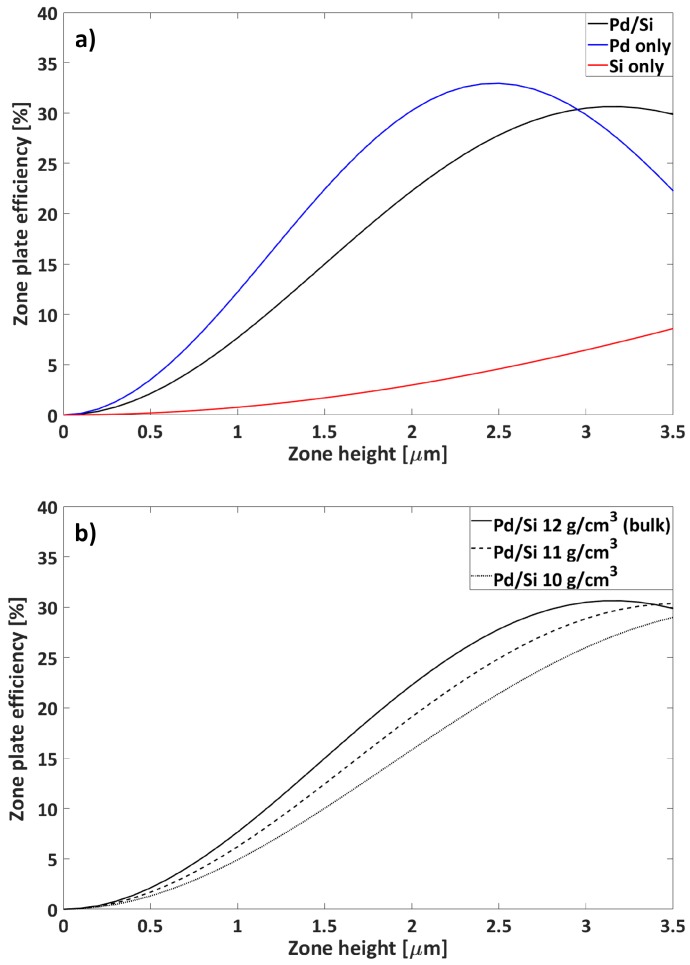
Zone plate efficiency at 9 keV as a function of zone height for (**a**) Pd/Si, Pd only, and Si only zone plates (bulk densities) and (**b**) Pd/Si zone plates with different Pd densities and bulk Si density. Calculations were done in MATLAB with GD-Calc.

A pure Si zone plate would require extreme thicknesses to serve directly as a zone plate device. At a 900 nm zone thickness at 9 keV, a first order diffraction efficiency of only 0.6% would be expected (Figure 4a). Oppositely, a pure Pd zone plate has a theoretical efficiency of 10% at the same thickness. A combined Pd/Si zone plate, as in our work, would show a shift in focusing performance so that thicker zones would be required to reach the maximum efficiency. The 30% maximum diffraction efficiency would be expected at a 3 μm thickness for a combined Pd/Si zone plate given a tabulated Pd density of 12 g/cm${}^{3}$. We kept the Si walls in our zone plates after filling with Pd due to the mechanical support that they provided. For a 900 nm thick Pd/Si zone plate with tabulated Pd density, an efficiency of 6.4% was expected.

The density of the ELD plated Pd was gravimetrically determined to be 10.7 ± 0.4 g/cm${}^{3}$. With lower Pd density, the expected zone plate efficiency also decreased (Figure 4b). It should be noted that a lower density than the tabulated value was expected for ELD plated Pd. Pores, voids, and impurities in the Pd deposit were common reasons for this. The inclusion of relatively light-weight elements in the ELD bath formulation, such as brighteners, would decrease the deposit density [30]. The expected efficiencies for 900 nm Pd/Si zone plates with Pd densities of 10 g/cm${}^{3}$ and 11 g/cm${}^{3}$ were 4.0% and 5.1%, respectively.

### 3.3. Focusing Performance

**Figure 5:**
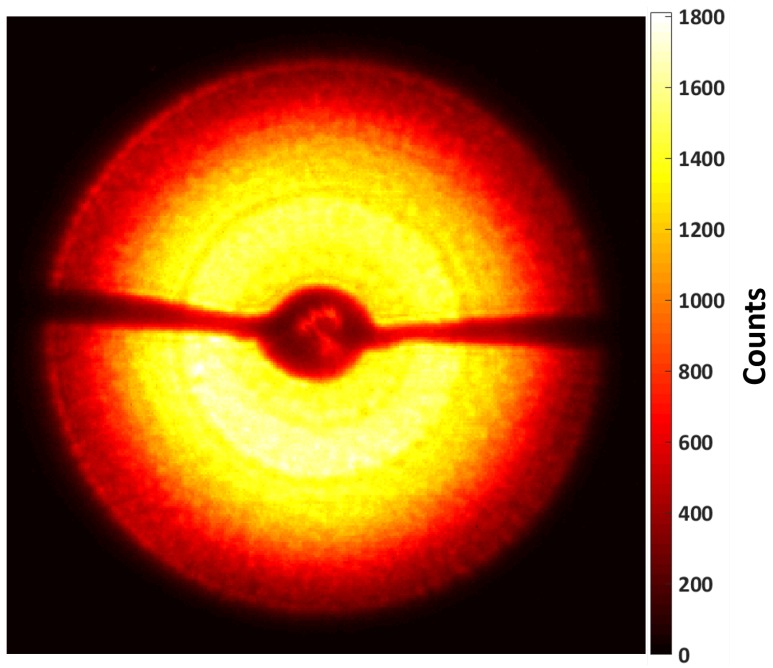
Raw image of the first order diffraction cone showing a map of the local zone plate efficiency.

Figure 5 shows an image of the first order diffraction cone and illustrates the local zone plate efficiency. The best measured total zone plate efficiency of our 900 nm thick devices at 9 keV was 1.9%. The main factor for the lower zone plate efficiency compared to theoretical values could be explained by a decrease of the local efficiency towards larger radii. The local efficiency was even to about 2/3 of the radius, and then gradually dropped outwards. This was believed to be due to the more tilted zones in the outer part (cf. Figure 3b). Moreover, the Au catalyst had a grid-like design with interconnects between the rings (Figure 2a). While this grid design was necessary to ensure zone verticality during the MACE process, the interconnects reduced the effective zone plate area. Lastly, the Pd overplating had a minor effect on the efficiency due to some absorption of the incoming photons.

## 4. Conclusions

In this paper, we presented a wet-chemical route to fabricate high aspect ratio Pd/Si zone plates aimed for the hard X-ray regime. MACE was used to fabricate a Si zone plate mold, and ELD was used to metalize the mold with Pd. We demonstrated and characterized the zone plates using this fabrication route. The optics device had a grid design with a 30 nm outermost zone width and a 900 nm zone thickness, thus an aspect ratio of 30:1. The lower zone plate efficiency of 1.9%, compared to theory, was mainly due to the tilt of the outermost zones, the loss of effective zone plate area by the grid design, and some photon absorption due to Pd overplating.

The MACE parameters used in this study were optimized for processing at ambient conditions with the grid zone plate design [20]. Vertically etched zones were obtained at the presented process conditions; however, some tilting was observed in the outermost parts. This was due to the mechanical instabilities of the free-standing Si structures with the grid design, and the aspect ratio was therefore limited to 30:1. To reach larger aspect ratios, a different zone plate design with interconnected Si structures should be adapted, as reported by Chang and Sakdinawat [17]. Combined with Pd electroless deposition, much higher efficiencies should be possible.

## Abbreviations

The following abbreviations are used in this manuscript:

MACE	Metal-assisted chemical etching
ALD	Atomic layer deposition
ED	Electrodeposition
ELD	Electroless deposition
EBL	Electron beam lithography
DI	Deionized
SEM	Scanning electron microscopy
FIB	Focused ion beam
OSA	Order sorting aperture
